# Quantum dot/glycol chitosan fluorescent nanoconjugates

**DOI:** 10.1186/s11671-015-0879-2

**Published:** 2015-04-10

**Authors:** Alexandra AP Mansur, Herman S Mansur

**Affiliations:** Center of Nanoscience, Nanotechnology and Innovation - CeNano2I, Department of Metallurgical and Materials Engineering, Federal University of Minas Gerais, Av. Antônio Carlos, 6627 - Escola de Engenharia, Bloco 2 - Sala 2233, Belo Horizonte, MG 31.270-901 Brazil

**Keywords:** 81.07.Ta, 78.67.Hc, 82.35.Pq, Glycol chitosan, Nanoparticle, Quantum dot, Colloid, Biopolymer, Bioconjugates, Nanomaterials

## Abstract

In this study, novel carbohydrate-based nanoconjugates combining chemically modified chitosan with semiconductor quantum dots (QDs) were designed and synthesised via single-step aqueous route at room temperature. Glycol chitosan (G-CHI) was used as the capping ligand aiming to improve the water solubility of the nanoconjugates to produce stable and biocompatible colloidal systems. UV-visible (UV–vis) spectroscopy, photoluminescence (PL) spectroscopy, and Fourier transform infrared (FTIR) spectroscopy were used to characterise the synthesis and the relative stability of biopolymer-capped semiconductor nanocrystals. The results clearly demonstrated that the glycol chitosan derivative was remarkably effective at nucleating and stabilising semiconductor CdS quantum dots in aqueous suspensions under acidic, neutral, and alkaline media with an average size of approximately 2.5 nm and a fluorescent activity in the visible range of the spectra.

## Background

Approximately 3 decades ago, quantum dots (QDs) emerged as a notable class of nanomaterials because of their unique set of optical, electronic, magnetic, and chemical properties [1-3]. Essentially, QDs are ultra-small semiconductor crystalline nanoparticles with size-dependent properties, which possess higher luminescence, narrower emission band, broader excitation wavelength range, and greater photostablility compared with fluorescent organic dyes [2]. Due to their small dimensions with extremely high surface area, these fluorescent nanocrystals must be stabilised by capping agents during their synthesis to restrict the growth of the nucleated nanoparticles [4]. Thus, QDs have been produced using a myriad of processes, such as entrapment in molecular films [5,6] and glasses [7,8], as well as encapsulation in polymer nanoparticles [9], organic solvents [10], and colloidal dispersions [11].

Since the seminal work of Murray et al*.* [12], the majority of QDs have been developed using organometallic routes at high temperature because they commonly result on monodisperse nanoparticles with high luminescent behaviour. However, water-soluble QDs have increasingly attracted the attention of the research community based on their potential use in biomedical and environmentally friendly applications [1,13,14]. Therefore, water-soluble polymers are a promising platform to develop innovative QD nanohybrids because they offer an attractive set of physicochemical properties associated with broad availability, large variety of chemical structures at relative low cost. In addition, polymers can be chemically functionalised and conjugated with other molecules for designed and specific applications [15-17]. Among the numerous alternative polymers for biomedical applications, chitosan (CHI) and its derivatives have often been selected due to their multidimensional properties [18,19]. However, chitosan is reasonably water-soluble only under acidic conditions, and it is practically insoluble at neutral and alkaline pH (at pH higher than its pKa ∼ 6.5), which significantly restricts its applications in medicine and biology at physiological pH (approximately 7.4). Hence, the chemical modifications of chitosan for producing water-soluble derivatives in a broader pH range, mainly under physiological conditions, are highly attractive for the preparation of nanohybrids and nanoconjugates for nanomedicine [9,20-25]. Surprisingly, only few reports have been published in the literature using chitosan and its derivatives as direct capping ligands for the synthesis of QDs in aqueous media [21-24].

Glycol chitosan (G-CHI) is a commercially available derivate of chitosan with improved hydrophilicity and biocompatibility and is frequently used in various biomedical applications such as drug delivery, siRNA carrier, cancer imaging, and therapy [26]. Interesting reports using G-CHI combined with nanomaterials (e.g. gold nanoparticles) have been published by Kim and collaborators [26,27], as well as studies on PEG-conjugated chitosan derivatives for the preparation of QDs [28]. Nevertheless, no study was found in the consulted literature addressing the direct synthesis of QDs using glycol chitosan as capping ligands by aqueous colloidal chemistry.

Thus, in this study, novel carbohydrate-based nanoconjugates combining glycol chitosan with CdS semiconductor QDs were designed and synthesised via a single-step aqueous process at room temperature. G-CHI was used as the capping ligand to produce water-soluble colloidal bioconjugates. The results demonstrated that the glycol chitosan derivative was effective at nucleating and stabilising luminescent CdS QDs in aqueous colloidal dispersions under acidic, physiological, and alkaline media, indicating considerable potential for biomedical and pharmaceutical applications in nanomedicine.

## Methods

### Materials

All of the reagents and precursors, including cadmium perchlorate hydrate (Sigma-Aldrich, St. Louis, MO, USA, Cd(ClO_4_)_2_ · 6H_2_O), sodium sulphide (Synth, Diadema, Brazil, >98%, Na_2_S · 9H_2_O), and hydrochloric acid (Sigma-Aldrich, St. Louis, MO, USA, 36.5% to 38%, HCl) were used as received. Glycol chitosan (G-CHI; Sigma-Aldrich, St. Louis, MO, USA, PN# G7753; degree of polymerization ≥400, lot supplied = 2,000 (*M*_w_ ~ 410 kDa); degree of deacetylation DD ≥60%, lot supplied = 76.2%) was used as the ligand. Chitosan (Aldrich Chemical, St. Louis, MO, USA, catalogue#419419; high molecular weight, *M*_w_ = 310 to >395 kDa; degree of deacetylation DD ≥75.0%; viscosity 800 to 2,000 cPoise, 1 wt.% in 1% acetic acid) was used as the reference polysaccharide ligand. Unless otherwise indicated, deionised water (DI water; Millipore Simplicity™, Millipore, Billerica, MA, USA) with a resistivity of 18 MΩ · cm was used to prepare the solutions, and the procedures were conducted at room temperature (RT; 23°C ± 2°C).

### Synthesis of CdS quantum dots

A chitosan solution (1%, *w*/*v*) was prepared by dispersing CHI powder in an aqueous solution (2%, *v*/*v*) of acetic acid. The mixture was placed under constant stirring overnight at room temperature, until complete solubilisation had occurred (pH ~ 3.6). Glycol chitosan solution (1.0%, *w*/*v*) was prepared by dissolving G-CHI powder in DI water under moderate magnetic stirring for 2 h until complete solubilisation had occurred (pH ~ 8.4). Before synthesising the CdS QDs, chitosan and glycol chitosan solutions were diluted with DI water to a concentration of 0.4 mg.mL^−1^ and the pH was adjusted with NaOH or HCl solutions (0.1 mol.L^−1^). CdS nanoparticles stabilised by G-CHI and CHI were synthesised via an aqueous route in a reaction flask at room temperature as previously described [29]: glycol chitosan solution or chitosan solution (0.4 mg.mL^−1^) was added to the flask. With moderate magnetic stirring, the Cd^2+^ (Cd(ClO_4_)_2_ · 6H_2_O, 0.75 mmol.L^−1^) and S^2−^ precursor solutions (Na_2_S · 9H_2_O, 0.37 mmol.L^−1^) were added to the reaction flask (S:Cd molar ratio was kept at 1:2) and stirred for 3 min. The obtained CdS QD dispersions were clear, homogeneous, and yellowish, which were referred to as CdS_G-CHI_5.0, CdS_G-CHI_7.4, CdS_G-CHI_8.4, and CdS_CHI_6.0 based on the capping ligand and pH used during the synthesis (5.0 ± 0.2, 7.4 ± 0.2, 8.4 ± 0.2, and 6.0 ± 0.2, respectively). The CdS QD colloids were dialyzed for 24 h (water changes after 2 and 4 h) against 3 L of DI water using a cellulose membrane with molecular weight cutoff (MWCO) of 14,000 Da with moderate stirring at room temperature. After purification, the QD dispersions were stored at 6°C ± 2°C for further use.

### Characterisation of the CdS quantum dots and capping agents

UV-visible (UV–vis) spectroscopy measurements were conducted using a PerkinElmer instrument (Lambda EZ-210, PerkinElmer, Waltham, MA, USA) in the transmission mode with samples in a quartz cuvette over a wavelength range of 600 to 190 nm. All of the experiments were conducted in triplicates (*n* = 3) unless specifically noted, and the data are presented as the mean ± standard deviation.

Photoluminescence (PL) characterisation of the CdS conjugates was conducted based on spectra acquired at room temperature using a violet diode laser module at *λ*_exc_ = 405 nm (150 mW, Roithner LaserTechnik, GmbH, Vienna, Austria) coupled to a USB4000 VIS-NIR spectrophotometer (Ocean Optics, Dunedin, FL, USA). All of the tests were performed using a minimum of four repetitions (*n* ≥ 4). Digital colour images were collected of the QD fluorescence in the visible range of the spectrum (cutoff filter 535 nm).

Nanostructural characterisations of the QD bioconjugates were based on the images and selected area electron diffraction (SAED) patterns obtained using a Tecnai G2-20-FEI transmission electron microscope (TEM; FEI, Hillsboro, OR, USA) at an accelerating voltage of 200 kV. Energy-dispersive X-ray (EDX) spectra were collected using the TEM for element chemical analysis. In all of the TEM analyses, the samples were prepared by dropping the colloidal dispersion onto a porous carbon grid. The QD size and size-distribution data were obtained based on the TEM images by measuring at least 100 randomly selected nanoparticles using an image processing program (ImageJ, version 1.44, public domain, National Institutes of Health).

X-ray diffraction (XRD) patterns were recorded using a PANalytical X´Pert diffractometer (PANalytical, Almelo, The Netherlands) (Cu-Kα radiation with *λ* = 1.5406 Å). Measurements were performed in the 2*θ* range from 6° to 70° with steps of 0.067°. For sample preparation, QD colloidal medium was concentrated and purified using an Amicon® Ultra Filter (Millipore, Billerica, MA, USA) with a 30,000 molecular mass (*M*_w_) cutoff cellulose membrane, dropped on a glass slide and dried in an oven at 40°C ± 1°C for 12 h.

Dynamic light scattering (DLS) and Zeta potential (ZP) analyses were performed using a Brookhaven ZetaPlus instrument (Brookhaven Instruments Corporation, Holtsville, NY, USA) with a laser light with wavelength of 660 nm (35-mW red diode laser) and a thermostat with temperature stabilisation. Standard square acrylic cells with a volume of 4.5 mL were used. Samples were measured at the temperature of 25°C ± 2°C, and the light scattering was detected at the angle of 90°. Before the DLS analysis of the QDs, the colloidal solutions (3 mL) were filtered three times through a 0.45-μm aqueous syringe filter (Millex LCR 25 mm, Millipore, Billerica, MA, USA) to remove any possible dust. Five measurements were obtained for each system and averaged. Zeta potential measurements were performed in the CdS QD colloidal aqueous solutions using the laser Doppler electrophoresis technique. All of the tests were performed using a minimum of ten replicates (*n* ≥ 10), and the values were averaged.

CdS quantum dots and ligands were analysed by the diffuse reflectance infrared Fourier transform spectroscopy (DRIFTS) method (Thermo Fisher Scientific, Waltham, MA, USA, Nicolet 6700) over the range from 4,000 to 400 cm^−1^ using 64 scans and a 4 cm^−1^ resolution. These samples were prepared by placing a droplet of the G-CHI solution, CHI solution, or CdS QD dispersions onto KBr powder and drying at 60°C ± 2°C for 24 h. Glycol chitosan as supplied was also pressed and analysed by attenuated total reflectance (ATR) over the range of 4,000 to 675 cm^−1^ using 32 scans and a 4 cm^−1^ resolution.

## Results and discussion

### Characterisation of CdS quantum dots capped by chitosan

#### UV–vis spectroscopy

**Figure 1:**
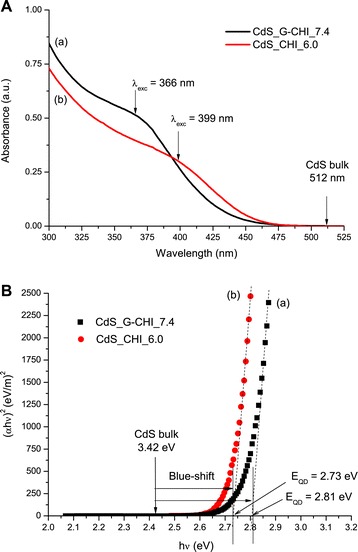
UV–vis spectroscopy analysis as a function of ligand. (**A**) UV–vis spectra and (**B**) optical band gap using “Tauc” relation of CdS_G-CHI_7.4 (a) and CdS_CHI_6.0 (b) conjugates.

Figure 1A presents the UV–vis spectroscopy results from the synthesis of semiconductor nanoparticles using glycol chitosan at physiological pH (7.4) as the stabilising ligand compared with chitosan. The pH of 6.0 was selected for the synthesis of the quantum dots with chitosan because this polymer is water-soluble at pH values lower than approximately 6.0 [30]. Under acidic conditions, the amine groups of chitosan are partially or completely protonated, resulting in the repulsion between chains, thereby favouring the chitosan-water interaction, which overcomes the associative forces between chains and promotes chitosan solubility.

The UV–vis absorption spectra of the CdS nanoconjugates exhibit a broad absorption band between 300 to 450 nm associated with the first excitonic transition (*λ*_exc_). This result indicates that CdS nanocrystals were synthesised within the ‘quantum confinement regime’ based on the energy blueshift observed for the curves compared with the ‘bulk’ value for the CdS semiconductor (Figure 1A, arrow at *λ* = 512 nm) [1]. The average sizes of CdS nanoparticles were determined using Henglein’s empirical model [31], which relates the diameter of the CdS nanoparticle (2*R*) to the exciton absorption transition onset (*λ*_exc_) in the UV–vis spectra according to Equation 1.

1 $$ 2R\left(\mathrm{nm}\right)=\frac{0.1}{0.1338-0.0002345*{\lambda}_{\mathrm{exc}}} $$

Based on the UV–vis spectra, the average sizes (diameter, 2*R*) were calculated as 2.1 ± 0.1 and 2.5 ± 0.1 nm for CdS_G-CHI_7.4 and CdS_CHI_6.0, respectively.

The optical band gap (absorbance onset, *E*_QD_) values of QDs extracted from the curves using the ‘Tauc relation’ [32] (Figure 1B) were 2.81 ± 0.02 and 2.73 ± 0.02 eV for QDs stabilised by glycol chitosan and chitosan, respectively. Because these band gap values (*E*_QD_) are higher than the reference bulk value (*Eg* = 2.42 eV) for CdS referred to as “blueshift” (i.e. *E*_QD_ − *Eg*), it can be stated that colloidal CdS QDs were effectively synthesised in aqueous media using glycol chitosan ligands at physiological conditions. To the best of our knowledge, there has been no reported literature in which bioconjugates based on CdS QDs were directly produced and stabilised by glycol chitosan as the capping ligand that were synthesised at room temperature using strictly water colloidal chemistry.

**Figure 2:**
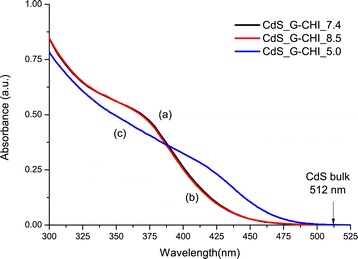
UV–vis spectroscopy analysis as a function of pH. UV–vis spectra for CdS_G-CHI_7.4 (a), CdS_G-CHI_8.5 (b), and CdS_G-CHI_5.0 (c).

The UV–vis results presented in Figure 2 also demonstrated that the glycol chitosan derivative was effective at nucleating and stabilising CdS QDs in aqueous suspensions in acidic (Figure 2(c), CdS_G-CHI_5.0), neutral (Figure 2(a), CdS_G-CHI_7.4), and alkaline (Figure 2(b), CdS_G-CHI_8.4) media.

Based on the spectra in Figure 2 and the results presented in Table 1, the pH significantly influences the formation/growth/stabilisation of the CdS QDs in the glycol chitosan colloidal solution. When the synthesis pH was raised from acidic to neutral and then alkaline, the nanocrystal size decreased. At pH = 5.0, some of the amine groups of glycol chitosan are protonated, forcing the positively charged transition metal to compete with a hydrogen ion for complexation with an amine electron pair (metal-ligand interactions). However, as the pH increases to 7.4 and 8.5, most of the amine groups in the glycol chitosan chain become available for dative bonding (electron donor) with Cd^2+^, thus reducing the electrostatic repulsion (Cd^2+^↔NH_3_^+^) and favouring the stabilisation of the CdS nanocrystals at smaller dimensions. The similarity of the QD parameters (*λ*_exc_, 2*R*, and *E*_QD_) measured in neutral and alkaline media indicates that the amine groups of G-CHI are fully deprotonated at pH 7.4 (pKa ~ 6.5).

**Table 1:** **Quantum dots parameters: excitonic transition wavelength, estimated particle diameter, band-gap energy, and blueshift**

**System**	**Ligand**	**pH**	**Parameter**	**Values**
CdS_G-CHI_7.4			*λ*_exc_ (nm)	369 ± 2
			2*R* (nm)	2.1 ± 0.1
	Glycol chitosan	7.4 ± 0.2	(CdS) (μmol.L^−1^)	4.4 ± 0.2
			*E*_QD_ (eV)	2.81 ± 0.02
			Blueshift (eV)	0.39 ± 0.02
CdS_G-CHI_5.0			*λ*_exc_ (nm)	413 ± 2
			2*R* (nm)	2.7 ± 0.1
	Glycol chitosan	5.0 ± 0.2	(CdS) (μmol.L^−1^)	1.3 ± 0.2
			*E*_QD_ (eV)	2.64 ± 0.02
			Blueshift (eV)	0.22 ± 0.02
CdS_G-CHI_8.5			*λ*_exc_ (nm)	367 ± 2
			2*R* (nm)	2.1 ± 0.1
	Glycol chitosan	8.5 ± 0.2	(CdS) (μmol.L^−1^)	4.4 ± 0.2
			*E*_QD_ (eV)	2.81 ± 0.02
			Blueshift (eV)	0.39 ± 0.02
CdS_CHI_6.0			*λ*_exc_ (nm)	399 ± 2
			2*R* (nm)	2.5 ± 0.1
	Chitosan	6.0 ± 0.2	(CdS) (μmol.L^−1^)	1.8 ± 0.2
			*E*_QD_ (eV)	2.73 ± 0.02
			Blueshift (eV)	0.31 ± 0.02

#### Photoluminescence spectroscopy analysis

**Figure 3:**
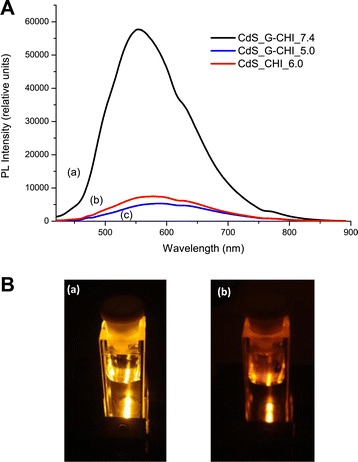
PL analysis of CdS conjugates. (**A**) PL spectra of CdS_G-CHI_7.4 (a), CdS_CHI_6.0 (b), and CdS_G-CHI_5.0. (**B**) Digital images of emission of CdS_G-CHI_7.4 (a) and CdS_CHI_6.0 (b).

Figure 3A shows the photoluminescence spectra of the nanohybrids synthesised with glycol chitosan (pH = 7.4 and pH = 5.0, Figure 3A(a) and Figure 3A(c)) and chitosan (pH = 6.0, Figure 3A(b)) collected at room temperature. The band edge (excitonic) emission was not detected, and the emissions in the green-red range arising from the intrinsic defects in the nanocrystals were observed for both of the systems, as reported in the literature [15,16,21,33-35]. The green luminescence is favoured by the synthesis of nanoparticles under an excess of metal cations (Cd^2+^) [33-35], compatible with the procedure used in this study [29] (molar ratio of cation:anion = 2:1). The orange luminescence arises from the interstitial atoms of the metal in the semiconductor lattice (Cd*i*), while the red band of PL is assigned to vacancy states (*V*_Cd_ − *V*_*S*_ or *V*_*S*_) [34,35]. In addition, note that the luminescent response of G-CHI QDs nanoparticles was approximately 7.5-fold higher than the measured value for CdS_CHI_6.0 system (Figure 3A). This behaviour can be qualitatively observed in the images showed in Figure 3B with the much stronger luminescent response of G-CHI QDs nanoparticles (image, a) compared to CHI QDs (image, b). As luminescence is directly proportional to nanoparticles concentration, it was expected that the emission of the CdS_G-CHI_7.4 system would be more intense compared with CdS_CHI_6.0 because the molar concentration of glycol QDs is approximately 2.5 times higher (Table 1). In contrast, because glycol chitosan nanoparticles were 20% smaller than chitosan stabilised nanocrystals, it is considered that the higher surface disorder, under-coordinated sites, and dangling bonds would dominate the luminescent properties, thus creating non-radiative pathways that dissipate quantum dot emissions, which resulted in the decreased PL intensity. The overall balance is an increased luminescence intensity that may also be favoured by the absence of protonated amines at pH 7.4 that can act as electron scavengers, quenching the photoluminescence. For the CdS_G-CHI_5.0 nanoconjugates, the intensity of the PL spectrum was relatively lower than the response from the CdS_CHI_6.0 sample, which was assigned to the higher number of protonated amine groups at lower pH, as the sizes of both nanoparticles were equivalent.

#### TEM morphological analysis

**Figure 4:**
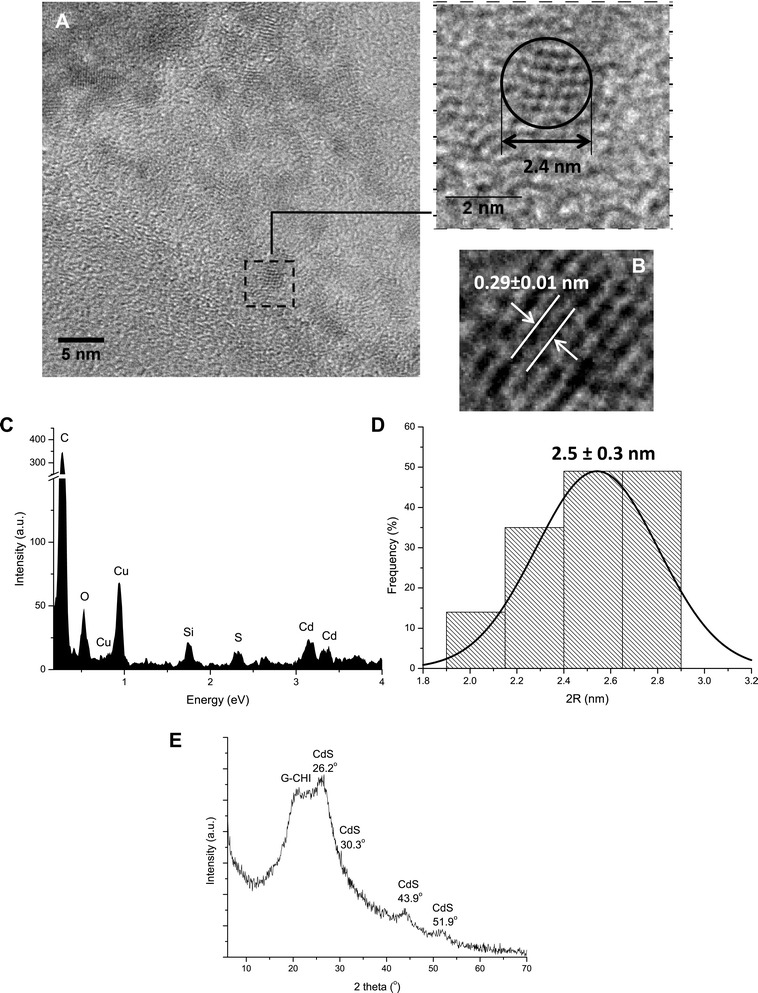
Morphological and structural analysis of CdS_G-CHI_7.4. (**A**) TEM image. (**B**) Nanocrystal plane spacing (SAED). (**C**) EDX spectrum. (**D**) Histogram of nanoparticle size distribution. (**E**) X-ray diffraction pattern.

In this study, the morphological and structural features of the CdS QDs were characterised using transmission electron microscopy (TEM) coupled to an EDX microprobe and using SAED analysis. Figure 4A shows a representative image of CdS QDs produced with glycol chitosan as the capping agent at pH = 7.4. Some of the CdS_G-CHI_7.4 nanoparticles are clustered, but the isolated particles showed an apparent spherical morphological feature. Figure 4B shows the SAED pattern of the CdS nanoparticles indicating that nanoparticles were crystalline in nature. The histogram of the CdS_G-CHI_7.4 size distribution (Figure 4D) indicates a relatively monodisperse distribution with an average size of 2.5 ± 0.3 nm in agreement with the values estimated by UV–vis spectroscopy in the previous sections (2*R* = 2.1 ± 0.1 nm). EDX spectrum shows the chemical analysis of the nanocrystals with Cd and S as the major elements (Figure 4C), excluding copper, oxygen, and carbon peaks related to the TEM grid and the polymer stabiliser, and Si from the detector. Thus, the TEM results demonstrated that the CdS QDs were properly stabilised by glycol chitosan under physiological conditions.

#### XRD analysis

The XRD pattern of the CdS_G-CHI_7.4 nanoconjugates (Figure 4E) presented a broad peak in the range of 20° to 25° associated with the semicrystalline structure of the polymer capping agent and four peaks centred at 2*θ* 26.2° (*d* = 0.34 nm), 30.3° (*d* = 0.29 nm), 43.9° (*d* = 0.21 nm), and 51.9° (d = 0.18 nm), which can be attributed to CdS with cubic lattice structure (JCPDS 89–0440). Some peak broadening in the XRD pattern is normally observed caused by the formation of ultra-small nanocrystals, i.e. CdS quantum dots, combined with overlapped scattering from the biopolymer ligands. These results were compatible with the lattice fringes of an inter-planar distance of approximately 0.29 ± 0.01 nm assigned to the (200) plane and revealed by the SAED pattern (Figure 4B).

#### DLS and ZP analyses

The DLS results showed that the ‘number-average’ diameters of CdS_G-CHI_7.4 and CdS_CHI_6.0 were 40.4 ± 3.3 and 16.6 ± 0.6 nm, respectively. These values are higher than the semiconductor nanoparticle sizes estimated using the UV–vis absorbance curves and TEM analysis (2.1 to 2.5 nm). This trend was expected because DLS values correspond to the ‘hydrodynamic diameter’ (*HD*) of the nanoconjugates in the colloidal media and include the organic shell (capping ligand) and the inorganic core (CdS nanocrystal), and they cannot be directly compared to the TEM results (‘dry’ morphological analysis) or the UV–vis measurements (energy of band gap absorption). The difference of dimensions between the conjugates stabilised by CHI and G-CHI is mostly associated with the capping ligand, and they can be explained based on the influence of the solvation layers and polyelectrolyte effects. At pH = 6.0 (CdS_CHI_6.0), hydrogen bonds and hydrophobic interactions between chitosan chains are more favourable once the number of –NH_3_^+^ species and the interchain repulsive electrostatic forces are reduced, promoting the formation of a more compact structure. On the other hand, the more hydrophilic ethylene glycol groups grafted to the chitosan derivative present higher interaction with aqueous medium with a consequent enlargement of the organic shell of the nanosystem measured by the *HD* values in the DLS assays.

The ZP values (*ζ*) of CdS_CHI_6.0 conjugates were almost neutral (0.0 ± 0.5 mV) indicating that the surface of these nanoparticles was mainly covered with CHI polymer. The surface charge of chitosan tends towards zero at pH values near the isoelectric point of chitosan (i.e. pH from 6.0 to 7.0), in agreement with the compact structure evaluated by the DLS assay. The positive values of ZP for CdS_G-CHI_7.4 nanoconjugates (+9.9 ± 3.3 mV) were influenced by the inorganic nanoparticle surface charge, which was expected to be positive as a result of the synthesis performed under the excess of cadmium cations, associated with the more ‘open’ structure of glycol chitosan shell. In addition, because the ZP results were above −30 mV and below +30 mV, CdS nanoconjugates were not predominantly stabilised by electrostatic balance but relied on the steric hindrance of the polymer shell to prevent close contact between the nanoparticles.

#### FTIR spectroscopy analysis

**Figure 5:**
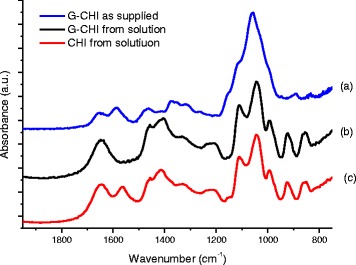
FTIR spectra of ligands. (a) Glycol chitosan as-supplied. (b) Glycol chitosan in solution (pH = 7.4 ± 0.2). (c) Chitosan in solution (pH = 6.0 ± 0.2).

The FTIR spectra of glycol chitosan as supplied and of glycol chitosan (pH = 7.4 ± 0.2) and chitosan (pH = 6.0 ± 0.2) after solubilisation are shown in Figure 5. The FTIR spectrum of chitosan presented absorption peaks at 3,400 to 3,200 cm^−1^ (OH stretching overlapped with NH stretching), 3,000 to 2,800 cm^−1^ (CH stretching), 1,650 to 1640 cm^−1^ (C = O stretching amide I), 1,590 to 1,560 cm^−1^ (NH bending, primary amine and amide II), and 1,450 to 1,380 cm^−1^ (CH bending). In addition, the absorptions at 1,020 to 1,050 cm^−1^ and 1,070 to 1,110 cm^−1^ indicate the C-O stretching vibrations in chitosan, which are associated with the C6-OH primary alcohol and the C3-OH secondary alcohol, respectively, and the bands at 1,160 and 996 cm^−1^ are assigned to the vibrations of the C-O-C saccharide bridge [36]. The modification of chitosan with the ethylene glycol (EG) moiety introduced chemical groups in the polymer chain (G-CHI) such as ether (C-O-C, 1,150 to 1050 cm^−1^), alcohol (−C-O, 1,260 to 1,000 cm^−1^), alkane (CH, 2,960 to 2,850 cm^−1^ and 1,450 to 1,300 cm^−1^), and hydroxyl (OH, 3,600 to 3,200 cm^−1^) [37,38], which mostly overlap the chitosan absorption bands. In addition to these bands, in the spectrum of the as-supplied glycol chitosan, the vibrations at 945 and 892 cm^−1^, associated with –CH_2_ rocking [37] and the –OC_2_H_4_ backbone [38], respectively, were observed. Note that the primary amine band of the as-supplied glycol chitosan was shifted from 1,560 to 1,590 cm^−1^ compared with chitosan. After solubilisation of glycol chitosan, this shift was higher, from 1,560 to 1618 cm^−1^ [39]. These shifts are related to the interactions between the hydrophilic OH groups from the EG moiety and NH from the primary amines of chitosan through hydrogen bonds.

**Figure 6:**
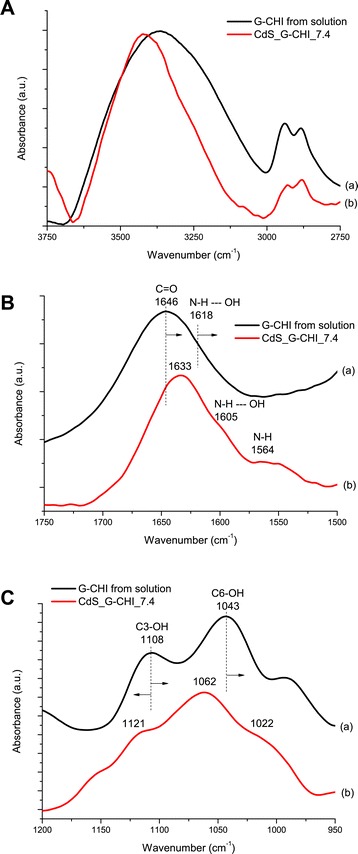
FTIR spectra of CdS-glycol chitosan QDs. (a) Glycol chitosan and (b) CdS_G-CHI_7.4. Vibrational spectrum region: (**A**) 3,750 to 3,250 cm^−1^; (**B**) 1,750 to 1,500 cm^−1^; (**C**) 1,250 to 950 cm^−1^.

After conjugating the quantum dots with the capping glycol chitosan biopolymer (curves (b) in Figure 6), several bands of G-CHI were observed in the FTIR spectra (curves (a) in Figure 6) that exhibited changes in their energies (i.e., wavenumber). These changes can be primarily attributed to the interactions occurring between the functional groups of the glycol chitosan ligand (amine/acetamide and hydroxyls) and the CdS QDs. The band of glycol chitosan at 3,400 to 3,200 cm^−1^ region (Figure 6A(a)), corresponding to the stretching vibration of the NH_2_ and OH groups, became significantly narrower after stabilisation of the CdS quantum dots (Figure 6A(b)). This peak narrowing indicates the reduction of ‘free’ amines groups after quantum dot stabilisation [40]. Additionally, in the FTIR spectrum of the G-CHI (Figure 6B(a)), the amide I band (at 1,646 cm^−1^) shifted to a lower wavenumber by 13 cm^−1^ for the CdS nanoconjugates (Figure 6B(b)). The band associated with the primary alcohol (C6-OH) vibration at 1,108 cm^−1^ was split into two bands at 1,121 and 1,162 cm^−1^, and the peak assigned to the C3-OH (secondary alcohol) stretching shifted its position to a lower wavenumber by 21 cm^−1^. In addition, the amine band was ‘redshifted’ (i.e., shifted to a lower wavenumber/energy) due to QD formation. It is believed that CdS nucleation and growth disrupts the hydrogen bonds –NH --- OH. Therefore, the band at 1,618 cm^−1^ shifted to 1,605 cm^−1^, and a band at 1,564 cm^−1^ related to the primary amine was observed. Based on the FTIR analyses, it was determined that the primary and secondary alcohols, amine, and acetamide (carboxyl) groups in glycol chitosan were involved in the stabilisation of the CdS_G-CHI nanoconjugates.

## Conclusions

In the present study, it is reported the direct synthesis of CdS QDs bio-functionalised by glycol chitosan using a single-step colloidal process at room temperature in acidic (pH = 5.0), physiological (pH = 7.4), and alkaline (pH = 8.4) aqueous media and compared to chitosan analogous system (pH = 6.0). The results indicated that the average diameter of the CdS nanoparticles using G-CHI ligand was larger at acidic conditions (pH = 5.0, 2*R* = 2.7 nm) than at neutral and alkaline solutions (2*R* = 2.1 nm at pH = 7.4 and pH = 8.4), predominantly associated with the stabilisation of conjugates by the deprotonation of the amine groups. In addition, the DLS results showed that the hydrodynamic diameters of CdS_G-CHI_7.4 and CdS_CHI_6.0 were 40.4 ± 3.3 and 16.6 ± 0.6 nm, respectively, attributed to the presence of the hydrophilic glycol groups in the chitosan chain leading to the expansion of the polymeric shell of the nanoconjugates by interactions with water molecules. Furthermore, the ZP results indicated that the colloidal stabilisation of CdS nanoconjugates relied on electrostatic interactions and steric hindrance of the polymer ligands to prevent aggregation or coalescence of the nanoparticles in aqueous solutions. Finally, it was demonstrated the feasibility of synthesising fluorescent CdS QDs in aqueous colloidal dispersions at physiological pH, indicating considerable potential to be used as biomarkers for biomedical and pharmaceutical applications in nanomedicine.
